# Novel polymorphisms in caspase-8 are associated with breast cancer risk in the California Teachers Study

**DOI:** 10.1186/s12885-015-2036-9

**Published:** 2016-01-12

**Authors:** Hannah Lui Park, Argyrios Ziogas, Jenny Chang, Bhumi Desai, Leona Bessonova, Chad Garner, Eunjung Lee, Susan L. Neuhausen, Sophia S. Wang, Huiyan Ma, Jessica Clague, Peggy Reynolds, James V. Lacey, Leslie Bernstein, Hoda Anton-Culver

**Affiliations:** Department of Epidemiology, University of California, Irvine, School of Medicine, 224 Irvine Hall, Irvine, CA 92697 USA; Keck School of Medicine, University of Southern California, Los Angeles, CA 90089 USA; Division of Cancer Etiology, Department of Population Sciences, Beckman Research Institute, City of Hope, Duarte, CA 91010 USA; Cancer Prevention Institute of California, Berkeley, CA 94704 USA

**Keywords:** Breast cancer, Single nucleotide polymorphism, *Caspase-8*

## Abstract

**Background:**

The ability of tamoxifen and raloxifene to decrease breast cancer risk varies among different breast cancer subtypes. It is important to determine one’s subtype-specific breast cancer risk when considering chemoprevention. A number of single nucleotide polymorphisms (SNPs), including one in *caspase-8* (*CASP8*), have been previously associated with risk of developing breast cancer. Because caspase-8 is an important protein involved in receptor-mediated apoptosis whose activity is affected by estrogen, we hypothesized that additional SNPs in *CASP8* could be associated with breast cancer risk, perhaps in a subtype-specific manner.

**Methods:**

Twelve tagging SNPs of *CASP8* were analyzed in a nested case control study (1,353 cases and 1,384 controls) of non-Hispanic white women participating in the California Teachers Study. Odds ratios (ORs) and 95 % confidence intervals (CIs) were calculated for each SNP using all, estrogen receptor (ER)-positive, ER-negative, human epidermal growth factor receptor 2 (HER2)-positive, and HER2-negative breast cancers as separate outcomes.

**Results:**

Several SNPs were associated with all, ER-positive, and HER2-positive breast cancers; however, after correcting for multiple comparisons (i.e., *p* < 0.0008), only rs2293554 was statistically significantly associated with HER2-positive breast cancer (OR = 1.98, 95 % CI 1.34-2.92, uncorrected *p* = 0.0005).

**Conclusions:**

While our results for *CASP8* SNPs should be validated in other cohorts with subtype-specific information, we conclude that some SNPs in *CASP8* are associated with subtype-specific breast cancer risk. This study contributes to our understanding of *CASP8* SNPs and breast cancer risk by subtype.

**Electronic supplementary material:**

The online version of this article (doi:10.1186/s12885-015-2036-9) contains supplementary material, which is available to authorized users.

## Background

Breast cancer risk factors include a woman’s age, family history, reproductive and gynecologic factors, and lifestyle factors including alcohol consumption and lack of physical activity [1]. When treating women at high risk for breast cancer, clinicians may recommend that women undergo increased screening, genetic testing, or chemoprevention [2–4]. Phase III breast cancer chemoprevention trials have now demonstrated the efficacy of selective estrogen receptor (ER) modulators (SERMs) (e.g., tamoxifen and raloxifene) and aromatase inhibitors in reducing the incidence of breast cancer. However, these drugs were significantly more effective at reducing the incidence of ER-positive breast cancer than ER-negative breast cancer [5–13]. ER-positivity is also associated with better prognosis after breast cancer diagnosis than ER-negativity [14, 15], while human epidermal growth factor receptor 2 (HER2)-positivity [16] and triple negativity (ER-negative, progesterone receptor (PR)-negative, and HER2-negative) [17] are each associated with worse prognosis. Drugs to target prevention of HER2-positive breast cancer and triple-negative breast cancers are also currently being studied [18]. With known undesirable side effects associated with chemopreventive medications that have been developed thus far, knowledge of one’s risk not only for any breast cancer but for specific subtypes of breast cancer would be helpful for a woman and her physician when considering chemopreventive therapy options.

Breast cancer risk models currently used by clinicians to identify women at high risk of developing breast cancer exhibit limited sensitivities and specificities [1]; and many studies have focused on identifying genetic variation associated with breast cancer risk with the hope that single nucleotide polymorphism (SNP) genotyping can be used to better stratify breast cancer risk and inform clinical management. While it is known that mutations in *BRCA1* and *BRCA2* markedly increase one’s risk of developing breast cancer [19, 20], a number of additional low and moderate-risk susceptibility variants have been identified, including one for *caspase-8* (*CASP8*), an enzyme involved in apoptosis [21].

Caspase-8 is activated in response to extrinsic apoptotic signals, including chemotherapy agents [22]. *In vitro*, estrogen inhibits caspase-8 activity and activity of other caspases [23]. The Breast Cancer Association Consortium (BCAC) has identified 3 SNPs in *CASP8*, namely rs1045485, rs17468277, and rs1830298, which are associated with breast cancer risk [24–26]. Other *CASP8* SNPs have shown to be associated with increased breast cancer risk [27–29]. Besides two BCAC studies, which found that rs1045485 was associated with a lower risk of PR-positive breast cancer [25], rs1830298 was associated with higher risk of ER-positive and triple-negative breast cancer [26], and rs36043647 was associated with lower risk of overall, ER-positive, ER-negative, and triple negative breast cancer [26], few studies have described associations between *CASP8* polymorphisms and subtype-specific breast cancer risk. Given the important role of caspase-8 in apoptosis, we hypothesized that additional *CASP8* polymorphisms would be associated with breast cancer risk and that the associations might be specific to some breast cancer subtypes. The aim of this study was to examine potential associations between 12 *CASP8* polymorphisms and breast cancer risk, overall and by subtype, using case and control samples nested within the California Teachers Study (CTS).

## Methods

### Ethics statement

This study was carried out in compliance with the Helsinki Declaration and approved by the Institutional Review Boards at each study center, namely, the City of Hope (COH), the University of Southern California (USC), the Cancer Prevention Institute of California (CPIC), the University of California at Irvine (UCI), and by the California State Committee for the Protection of Human Subjects, in accordance with assurances filed with and approved by the US Department of Health and Human Services. All study participants provided written informed consent to participate in the study.

### Participants

The CTS is a well-established prospective cohort study of 133,479 female California public school teachers and administrators who were enrolled in the California State Teachers Retirement System. A detailed account of the methods employed by the CTS has been published previously [30]. Briefly, participants completed a baseline questionnaire and returned it by mail in 1995–1996. The baseline survey, which collected information on demographics, personal and family cancer history, height, weight, history of hormone use, and behavioral factors including physical activity and alcohol consumption, is available on the CTS website (www.calteachersstudy.org). New diagnoses of first primary invasive breast cancer among cohort members were identified through annual linkages with California Cancer Registry (CCR), a legally mandated statewide population-based cancer reporting system in which cancer data are obtained from cancer patients’ pathology reports at the hospital in which the patient was initially diagnosed. CCR ascertainment of newly diagnosed cancers is estimated to be 99 % complete [31].

For this nested, breast cancer case control study, biospecimens were collected between 2005-2009 from breast cancer cases diagnosed under age 80 years and unaffected controls in the cohort, all of whom had continued residence in California during the study period (1995 to time of blood draw). Cases were women who had a histologically confirmed invasive first primary carcinoma of the breast (International Classification of Disease for Oncology code C50 restricted to morphology codes under 8590) after 1998. Unaffected control participants were selected from the cohort and frequency matched to the cases based on age at baseline (within 5-year age groups), self-reported race/ethnicity (white, African American, Latina, Asian, and other), and three broad geographic regions in California (surrounding the three CTS specimen collection centers: CPIC, USC/COH, and UCI).

### Collection of biological specimens and DNA extraction

The collection of specimens has been described previously [32]. Briefly, cases and controls provided a blood sample and completed a brief questionnaire at the time of blood draw, which updated breast and reproductive and gynecologic history and several lifestyle factors. Women who declined providing blood provided saliva in Oragene DNA self-collection kits (DNA Genotek, Kanata, ON, Canada). All biological specimens were sent overnight to the UCI laboratory. DNA was extracted from blood clots using Qiagen Clotspin Baskets and DNA QIAmp DNA Blood Maxi Kits (Qiagen, Inc., Valencia, CA, USA) in accordance with Qiagen protocols. DNA was extracted from saliva samples using the Oragene protocol (DNA Genotek).

### Genotyping

The 12 tagging SNPs included in this analysis were selected to capture all common linkage disequilibrium tagging SNPs [minor allele frequency (MAF) of at least 5 %], 20 kb upstream of the 5' untranslated region (UTR) and 10 kb downstream of the 3' UTR, in individuals of European ancestry with minimum pairwise *r*^*2*^ of at least 0.80, using data from the International HapMap Project for the white CEPH (Utah residents with ancestry from northern and western Europe) population [HapMap release 21, July 2006, genotype build 36 (http://hapmap.ncbi.nlm.nih.gov)] [32].

DNA samples from 1,751 cases and 1,697 controls were plated for genotyping. A random sample of 193 duplicates (105 cases and 88 controls) was included for quality control. The samples were genotyped using the Illumina Golden Gate Assay (Illumina, Inc., San Diego, CA USA) at the University of Southern California Core Facility. Twelve haplotype-tagging SNPs in *CASP8* were included and genotyped. Samples with genotype call rates <90 % were excluded. Among the remaining samples, 160 randomly selected duplicates exhibited a genotype concordance rate of 99.9 %. Additional details were described previously [32]. Because the majority of participants were non-Hispanic whites, we restricted analyses to 2,737 non-Hispanic white women (1,353 cases and 1,384 controls).

### Statistical analyses

All statistical tests were two-sided. We used unconditional logistic regression models to estimate the odds ratios (ORs), 95 % confidence intervals (CIs), and *p*-values for the association of invasive breast cancer and each SNP, using log-additive models. Allele frequencies are shown in Additional file 1: Table S1. We adjusted for potential confounding by study center and other known risk factors, namely, age at baseline, family history (having a first-degree relative with history of breast cancer), body mass index (<25, 25.0-29.9, ≥30 kg/m^2^), alcohol consumption in the past year (none, <20 g/day, ≥20 g/day), physical activity in the past 3 years (0-0.5 hrs/wk/yr, 0.51–4.0 hr/wk/yr, >4.0 hr/wk/yr), and menopausal and hormone therapy (HT) status (premenopausal, postmenopausal and never used HT, postmenopausal and used HT in the past, postmenopausal and using estrogen only at baseline, postmenopausal and using estrogen and progesterone at baseline, and unknown) at baseline. To potentially improve power by increasing subgroup homogeneity, we stratified our analysis by estrogen receptor (ER) and human epidermal receptor (HER2) status of breast cancer. We evaluated the association for ER-positive (*n* = 1,046), ER-negative (*n* = 155), HER2-positive (*n* = 159), and HER2-negative (*n* = 662) subtype. Some breast cancers were not included in any specific receptor (ER or HER2) subtype analysis because they were missing either ER or HER2 status. PR status was not included since PR expression usually follows ER expression [33] and the clinical rationale to determine associations with PR-specific breast cancer risk was lacking since no chemotherapies or preventive therapies are being studied for PR status-specific subtypes. While therapies targeting triple-negative breast cancer are being considered, the number of triple-negative cancers in our subset of cases and controls was too small for analysis (*n* = 60). We used the conservative Bonferroni correction to correct for multiple testing (*n* = 60, 12 SNPs x 5 outcomes). Statistical significance was set to *p* < 0.0008. All analyses were done using SAS software version 9.2.

Recombination rates and linkage disequilibrium across the *CASP8* gene was evaluated using the HapMap database (http://hapmap.ncbi.nlm.nih.gov) and r^2^ values were computed from the pairwise SNP genotype counts of the generated genotype data.

## Results

Baseline characteristics of the cases and controls are provided in Table 1. Consistent with other studies, family history of breast cancer, menopause and hormone therapy (HT) use, physical inactivity, and alcohol use were associated with breast cancer risk. Genotype distributions are provided in Additional file 1: Table S1.

**Table 1:** Selected baseline characteristics of study participants by case (invasive breast cancer) and control status

Variables	Cases (*n* = 1353)	%	Controls (*n* = 1384)	%	Chi square *p*-value
Age, years (mean ± SD)	55.0 ± 9.4		56.1 ± 9.5		
First-degree family history of breast cancer					0.0046
ᅟNo	1086	80.3	1158	83.7	
ᅟYes	237	17.5	187	13.5	
Body mass index, kg/m^2^					0.21
ᅟ < 25	779	57.6	759	54.8	
ᅟ25 to 29.9	384	28.4	385	27.8	
ᅟ ≥ 30	160	11.8	192	13.9	
Age at menarche, years					0.45
ᅟ < 13	700	51.7	695	50.2	
ᅟ ≥ 13	638	47.2	671	48.5	
Parity					0.12
ᅟ0	289	21.4	280	20.2	
ᅟ1	173	12.8	165	11.9	
ᅟ2	481	35.6	476	34.4	
ᅟ3	275	20.3	277	20.0	
ᅟ ≥ 4	120	8.9	165	11.9	
Age at first full-term pregnancy, years					0.38
ᅟ < 21	89	8.5	96	8.9	
ᅟ21-24	325	31.0	343	31.7	
ᅟ25-29	415	39.6	451	41.6	
ᅟ30-34	169	16.1	155	14.3	
ᅟ ≥ 35	51	4.9	38	3.5	
Hormone therapy (HT) at baseline					0.0001
ᅟPremenopausal	364	26.9	346	25.0	
ᅟPostmenopausal - never used HT	113	8.4	150	10.8	
ᅟPostmenopausal - past use HT	76	5.6	115	8.3	
ᅟPostmenopausal - current estrogen use	200	14.8	249	18.0	
ᅟPostmenopausal - current estrogen + progestin use	402	29.7	335	24.2	
ᅟUnknown	198	14.6	189	13.7	
Strenuous or moderate physical activity, during 3 years before baseline					0.0035
ᅟ0-0.50 hrs/week/year	290	21.4	289	20.9	
ᅟ0.51-4.00 hrs/week/year	632	46.7	572	41.3	
ᅟ4.01-24 hrs/week/year	424	31.3	514	37.1	
Grams per day of alcohol, during year before baseline					0.0006
ᅟNondrinkers	334	24.7	379	27.4	
ᅟ < 20 g/d	806	59.6	836	60.4	
ᅟ > =20 g/d	165	12.2	109	7.9	

Table does not list small percentages of missing values for some factors

### *CASP8* polymorphisms and invasive breast cancer risk

The adjusted ORs and 95 % CIs of overall invasive breast cancer with *CASP8* polymorphisms are shown in Table 2. Four SNPs had a *p*-value < 0.05 for positive associations with overall breast cancer (rs11899004, rs3769825, rs6723097 and rs6736233). The SNP most strongly associated with overall breast cancer risk was rs6736233, which conferred an OR of 1.38 (95 % CI 1.12-1.71, *p* = 0.0028) (Table 2). After correcting for multiple comparisons, none of the SNPs tested remained statistically significant at *p* < 0.0008.

**Table 2:** Multivariate adjusted odds ratios (OR) and 95 % confidence intervals (CI) of overall invasive breast cancer associated with caspase-8 polymorphisms

SNP	Position on Chr. 2	Major allele	Minor allele	MAF (controls)	Overall							
					Unadjusted				Adjusted^a^			
					OR	95 % CI		p**	OR	95 % CI		p**
rs12693932	202093395	C	T	0.47	1.042	0.936	1.160		1.050	0.950	1.180	
rs6745051	202108741	C	A	0.48	1.039	0.933	1.156		1.050	0.940	1.170	
rs3769825	202111380	G	A	0.45	1.097	0.986	1.219		1.120	1.010	1.250	0.034
rs11899004	202114026	G	A	0.14	1.162	1.002	1.348	0.048	1.170	1.010	1.360	0.041
rs6736233	202118974	G	C	0.06	1.367	1.111	1.682	0.003	1.380	1.120	1.710	0.003
rs1861270	202126615	G	A	0.27	1.036	0.921	1.164		1.070	0.950	1.200	
rs6723097	202128618	C	A	0.38	1.131	1.015	1.259	0.026	1.170	1.050	1.310	0.005
rs2293554	202131587	T	G	0.07	1.164	0.950	1.427		1.190	0.970	1.470	
rs1045485	202149589	G	C	0.11	1.036	0.875	1.226		1.020	0.860	1.210	
rs1035140	202152491	A	T	0.46	1.030	0.927	1.143		1.050	0.940	1.170	
rs700636	202153252	C	A	0.43	1.051	0.944	1.169		1.080	0.970	1.210	
rs11679181	202162338	C	T	0.44	0.944	0.848	1.050		0.920	0.830	1.030	

^**a**^Per-allele ORs. Models were adjusted for center, age, family history, BMI, recent physical activity, alcohol consumption, and menopause/HT status

**Only uncorrected *p* values <0.05 are listed

When ER-positive and ER-negative breast cancer outcomes were analyzed separately, the trends of increased risk with rs3769825, rs6723097 and rs6736233 as seen for overall breast cancer remained for ER-positive breast cancers (Table 3). However, after correcting for multiple comparisons, none of the associations remained statistically significant. None of the SNPs tested were associated with ER-negative breast cancer risk.

**Table 3:** Multivariate adjusted odds ratios (OR) and 95 % confidence intervals (CI) of ER-positive and ER-negative invasive breast cancer associated with caspase-8 polymorphisms

SNP	ER-positive								ER-negative							
	Unadjusted				Adjusted^a^				Unadjusted				Adjusted^a^			
	OR	95 % CI		p**	OR^**a**^	95 % CI		p**	OR	95 % CI		p**	OR^**a**^	95 % CI		p**
rs12693932	1.075	0.957	1.207		1.090	0.970	1.220		1.013	0.800	1.282		1.010	0.800	1.290	
rs6745051	1.072	0.955	1.203		1.080	0.960	1.220		0.994	0.784	1.259		0.990	0.780	1.260	
rs3769825	1.108	0.989	1.242		1.130	1.010	1.270	0.035	1.107	0.876	1.398		1.140	0.900	1.440	
rs11899004	1.160	0.990	1.358		1.170	1.000	1.380		1.029	0.734	1.443		1.030	0.730	1.460	
rs6736233	1.364	1.096	1.697	0.005	1.360	1.090	1.710	0.006	1.180	0.740	1.882		1.260	0.780	2.020	
rs1861270	1.038	0.914	1.178		1.070	0.940	1.210		1.066	0.824	1.379		1.110	0.860	1.450	
rs6723097	1.123	0.999	1.262		1.160	1.030	1.310	0.014	1.089	0.859	1.381		1.150	0.900	1.460	
rs2293554	1.137	0.915	1.413		1.170	0.930	1.460		1.162	0.747	1.806		1.210	0.770	1.900	
rs1045485	1.088	0.911	1.300		1.060	0.890	1.270		0.942	0.638	1.391		0.940	0.630	1.400	
rs1035140	1.025	0.916	1.147		1.040	0.930	1.170		1.028	0.815	1.297		1.080	0.860	1.370	
rs700636	1.021	0.910	1.144		1.050	0.940	1.180		1.046	0.828	1.322		1.100	0.870	1.400	
rs11679181	0.955	0.851	1.071		0.940	0.840	1.060		0.978	0.772	1.238		0.920	0.720	1.170	

^**a**^Per-allele ORs. Models were adjusted for center, age, family history, BMI, recent physical activity, alcohol consumption, and menopause/HT status

**Only uncorrected *p* values <0.05 are listed

Three of the four SNPs that were associated with overall invasive breast cancer (*p* value < 0.05) were associated with HER2-positive invasive breast cancer (rs11899004, rs6723097, and rs6736233). rs2293554 was also associated with HER2-positive invasive breast cancer (OR = 1.98, 95 % CI 1.34-2.92, uncorrected *p* = 0.0005). After correcting for multiple comparisons, rs2293554 was the only SNP that remained statistically significant. Two of the four SNPs that were associated with overall invasive breast cancer (*p* value < 0.05) were associated with HER2-negative invasive breast cancer (rs3769825 and rs6723097). However, after correcting for multiple comparisons, neither remained statistically significant (Table 4)

**Table 4:** Multivariate adjusted odds ratios (OR) and 95 % confidence intervals (CI) of HER2-positive and HER2-negative invasive cancer associated with caspase-8 polymorphisms

SNP	HER2-positive								HER2-negative							
	Unadjusted				Adjusted^a^				Unadjusted				Adjusted^a^			
	OR	95 % CI		p**	OR*	95 % CI		p**	OR	95 % CI		p**	OR*	95 % CI		p**
rs12693932	1.111	0.879	1.404		1.110	0.870	1.410		1.123	0.983	1.281		1.150	1.000	1.320	
rs6745051	1.148	0.908	1.450		1.150	0.900	1.460		1.095	0.959	1.249		1.120	0.980	1.280	
rs3769825	1.153	0.915	1.455		1.180	0.930	1.490		1.164	1.020	1.327	0.024	1.200	1.050	1.370	0.008
rs11899004	1.680	1.259	2.241	0.0004	1.620	1.210	2.180	0.0014	1.118	0.930	1.344		1.130	0.940	1.360	
rs6736233	1.959	1.332	2.881	0.001	1.890	1.270	2.810	0.0017	1.283	0.995	1.655		1.290	0.990	1.670	
rs1861270	0.983	0.758	1.275		1.050	0.800	1.370		1.098	0.952	1.268		1.150	0.990	1.330	
rs6723097	1.336	1.057	1.688	0.015	1.410	1.110	1.800	0.0055	1.170	1.025	1.337	0.020	1.220	1.070	1.400	0.004
rs2293554	1.945	1.341	2.822	0.001	1.980	1.340	2.920	**0.0005**	1.165	0.910	1.490		1.200	0.930	1.550	
rs1045485	0.852	0.572	1.268		0.810	0.540	1.220		1.072	0.872	1.317		1.030	0.830	1.270	
rs1035140	0.958	0.760	1.207		1.000	0.790	1.270		1.081	0.950	1.231		1.100	0.970	1.260	
rs700636	1.012	0.802	1.276		1.080	0.850	1.380		1.076	0.944	1.226		1.110	0.970	1.270	
rs1679181	1.048	0.831	1.323		0.990	0.780	1.260		0.910	0.798	1.039		0.890	0.780	1.020	

^**a**^Per-allele ORs. Models were adjusted for center, age, family history, BMI, recent physical activity, alcohol consumption, and menopause/HT status

**Only uncorrected *p* values <0.05 are listed; after correcting for multiple comparisons, only rs2293554 was statistically significantly associated with HER2-positive breast cancer risk

.

In summary, after correction for multiple testing, one of the twelve *CASP8* SNPs tested in our study remained nominally statistically significantly associated with invasive breast cancer, specifically, HER2-positive breast cancer.

### Linkage disequilibrium

An analysis of data from the HapMap database indicated that very low historical genetic recombination exists across the entire *CASP8* gene in individuals of European descent, with pairwise *D’* values near 1.0 for all SNP pairs spanning the gene in the database. The alleles at the five markers that were associated with breast cancer risk in this study before correcting for multiple comparisons were not strongly correlated, as measured by the linkage disequilibrium measure *r*^*2*^. This low correlation (*r*^*2*^) in the context of high linkage disequilibrium (*D’*) was expected given that the SNPs were selected as tagging markers. Three pairs of SNPs showed *r*^*2*^values greater than 0.4: *r*^*2*^ = 0.44 for rs11899004 and rs2293554; *r*^*2*^ = 0.52 for rs11899004 and rs6736233; and *r*^*2*^ = 0.45 for rs3769825 and rs6723097. The remaining pairwise *r*^*2*^ values were all less than 0.2. rs6723097 and rs6736233 were the two SNPs most significantly associated with breast cancer risk overall, with uncorrected *p*-values of 0.0053 and 0.0028, respectively. These two SNPs are uncorrelated (*r*^*2*^ = 0.07) and likely represent independent associations.

## Discussion

This study is the first to identify the *CASP8* SNP, rs2293554, to be statistically significantly associated with HER2-positive breast cancer risk in non-Hispanic white women. In our study, the observed OR of 1.98 with 95 % confidence interval of 1.34–2.92 for HER2-positive breast cancer risk was surprisingly high, especially given the small number of HER2-positive breast cancers in our study. It is possible that the observation may have been due to chance. A previous study reported that rs2293554 was not associated with breast cancer risk overall [34], similar to what we observed here; however, subtype-specific breast cancers were not evaluated in that study.

The most recent BCAC paper on *CASP8* [26] covered the analysis of 501 typed and 1232 imputed SNPs, and, while some CTS samples were included in the BCAC study, there was only overlap of 57 triple-negative and 49 controls between the BCAC study and our present analysis. rs2293554 was not included on the panel of *CASP8* SNPs analyzed in the BCAC paper [26]; however, using the SNP lookup function on the BCAC website (http://apps.ccge.medschl.cam.ac.uk/consortia/bcac), we found that rs2293554 was not associated with overall, ER+, or ER- breast cancer risk. Data for HER2-specific breast cancer risk were not available on the website, but through personal email communication with the BCAC Data Manager, we learned that the BCAC data indicated that there was not an association between rs2293554 and HER2-positive breast cancer risk. rs2293554 was in strong LD with 16 of the 109 SNPs identified in the BCAC paper to be associated with overall breast cancer risk with FDR < 0.05 [26], with r^2^ > 0.50, according to the Linkage Disequilibrium Calculator (https://caprica.genetics.kcl.ac.uk/~ilori/ld_calculator.php), using the European panel in the 1000 genomes project; however, their effects were in the opposite direction (Additional file 2: Table S2). While our observation was not consistent with those in the BCAC study, our data demonstrates that SNPs can have different associations with breast cancer risk according to subtype and that rs2293554, with its nominally significant association with HER2-positive breast cancer risk in the CTS cohort, warrants further investigation.

Our study confirmed results from a meta-analysis, in which rs6723097 was associated with increased breast cancer risk [OR = 1.16 (95 % CI 1.07–1.25)] [34], and from a separate study [OR = 1.15 (95 % CI 1.01–1.30)] [27]. Here, the observed OR was 1.17 (95 % CI 1.05–1.31). Also consistent with previous studies, no associations with breast cancer risk were found for rs1035140 [34] and rs1861270 [27]. Eleven of the 12 SNPs analyzed in our study were included in a recent fine-mapping analysis by the BCAC [26]. Their findings were consistent with ours in that the 11 SNPs were not statistically significant after adjusting for multiple comparisons, or, in the case of the other paper, genome-wide significance of *P* = 5 x 10^-8^. The results for these SNPs were not shown by receptor subtype. To correct for multiple testing, we used Bonferroni adjustment, which is very conservative, since the SNPs and phenotypes we tested were somewhat correlated. Given the importance of replicating genetic associations [35], our study, conducted in a well-established, well-characterized prospective cohort [30] contributes important information on the relationship between *CASP8* polymorphisms and breast cancer risk.

Our results for rs1045485 were not consistent with those from two meta-analyses, which reported inverse associations with breast cancer, with pooled ORs of 0.87 (95 % CI 0.83-0.92) [28] and 0.79 (95 % CI 0.69-0.92) [29]. Our findings are consistent with a number of independent studies on the same SNP, some of which were included in the meta-analyses [28, 29] and a separate study [34] in which no association was found between this SNP and breast cancer risk. The MAF (10.5 % ) we observed in this study (all non-Hispanic Whites) is similar to that seen in the women of European ancestry [10, 35]. One of the BCAC studies on *CASP8*, which involved >30,000 invasive breast tumors, showed that rs1045485 was most strongly related with the risk of PR-negative tumors [25], but an association was not replicated in a later BCAC study [26]. Because no reports of development of PR status-specific chemoprevention were found at the time of the study, PR-specific subtypes were not included as outcomes in this study.

While the polymorphic *CASP8* sites identified in this study are all intronic, it is possible that they may affect expression of the protein or RNA splicing, which may affect protein-protein interactions and other functions. rs6723097 and rs6736233 were found to have features consistent with involvement in gene transcription regulation according to the Variant Effect Predictor (VEP) on the Ensembl website (http://uswest.ensembl.org/Homo_sapiens/Tools/VEP) [36]. The other SNPs we found to be associated with breast cancer risk did not have such features. However, rs12693932 and rs6745051 are in strong LD with each other, and they are also in strong LD with the SNP rs13006529, which is a missense, according to the University of Washington Genome Variation Server (http://gvs.gs.washington.edu/GVS144/). Also, rs1861270 is in strong LD with the SNP rs3769823, which is also a missense. Neither rs13006529 nor rs3769823 have been reported to be associated with breast cancer risk. The remaining SNPs on our panel are not in LD with other SNPs with known functions.

## Conclusions

We conclude that the *CASP8* SNP, rs2293554, is nominally statistically significantly associated with HER2-positive breast cancer risk in non-Hispanic white women, even after stringent correction for multiple comparisons. Other *CASP8* SNPs were also associated with overall, ER-positive, and HER2-negative breast cancer risk but the associations were not statistically significant after correction. While our results should be validated in other cohorts with subtype-specific information, this study contributes to our understanding of *CASP8* SNPs and subtype-specific breast cancer risk. The mechanistic and functional consequences of *CASP8* SNPs in breast cancer development and their relevance in women of other racial/ethnic groups remain to be investigated.

## Additional files

Additional file 1:
**Table S1.** Distributions of *CASP8* SNPs in controls and cases, overall and by subtype. Samples in which genotype was unknown are not included in this table. (DOC 88 kb) Additional file 2:
**Table S2.** SNPs from Lin et al. [26] that are in strong LD (r^2^ > 0.5) with rs2293554. *Odds Ratio (OR), Lower Confidence Limit (LCL), and Upper Confidence Limit (UCL) as reported in Lin et at. [26]. (DOC 44 kb)

## Abbreviations

SNP: Single nucleotide polymorphism
*CASP8*: *caspase-8*
ER: Estrogen receptor
HER2: Human epidermal growth factor receptor 2
CTS: California Teachers Study
HT: Hormone therapy
