# Supplementary material for: Novel polymorphisms in caspase-8 are associated with breast cancer risk in the California Teachers Study
Source: BMC Cancer. 2016 Jan 12;16:14. doi: 10.1186/s12885-015-2036-9 (PMC4711015; doi:10.1186/s12885-015-2036-9)
Supplement: Additional file 1: — Table S1. Distributions of CASP8 SNPs in controls and cases, overall and by subtype. Samples in which genotype was unknown are not included in this table. (DOC 88 kb) [file 12885_2015_2036_MOESM1_ESM.doc]

| Supplementary Table 1. Distributions of *CASP8* SNPs in controls and cases, overall and by subtype. | | | | | | | | | | | |  |  |
| --- | --- | --- | --- | --- | --- | --- | --- | --- | --- | --- | --- | --- | --- |
| **SNP** | **Genotype** | **Controls (n=1384)** | **%** | **Cases, overall (n=1353)** | **%** | **Cases, ER+ (n= 1046)** | **%** | **Cases, ER- (n= 155)** | **%** | **Cases, HER+ (n= 159)** | **%** | **Cases, HER- (n= 662)** | **%** |
| rs12693932 |  |  |  |  |  |  |  |  |  |  |  |  |  |
|  | CC | 387 | 28.0 | 358 | 26.5 | 260 | 24.9 | 46 | 29.7 | 39 | 24.5 | 164 | 24.8 |
|  | TC | 697 | 50.4 | 693 | 51.4 | 554 | 53.1 | 72 | 46.5 | 83 | 52.2 | 337 | 51.1 |
|  | TT | 298 | 21.6 | 298 | 22.1 | 229 | 22.0 | 37 | 23.9 | 37 | 23.3 | 159 | 24.1 |
| rs6745051 |  |  |  |  |  |  |  |  |  |  |  |  |  |
|  | AA | 310 | 22.4 | 308 | 22.9 | 239 | 22.9 | 36 | 23.5 | 40 | 25.3 | 159 | 24.2 |
|  | AC | 693 | 50.2 | 688 | 51.2 | 548 | 52.6 | 73 | 47.7 | 81 | 51.3 | 336 | 51.1 |
|  | CC | 378 | 27.4 | 349 | 25.9 | 255 | 24.5 | 44 | 28.8 | 37 | 23.4 | 162 | 24.7 |
| rs3769825 |  |  |  |  |  |  |  |  |  |  |  |  |  |
|  | AA | 280 | 20.2 | 305 | 22.6 | 231 | 22.1 | 39 | 25.2 | 37 | 23.3 | 155 | 23.4 |
|  | AG | 681 | 49.2 | 663 | 49.1 | 527 | 50.5 | 69 | 44.5 | 80 | 50.3 | 333 | 50.4 |
|  | GG | 422 | 30.5 | 381 | 28.2 | 285 | 27.3 | 47 | 30.3 | 42 | 26.4 | 173 | 26.2 |
| rs11899004 |  |  |  |  |  |  |  |  |  |  |  |  |  |
|  | AA | 27 | 2.0 | 38 | 2.8 | 33 | 3.2 | 1 | 0.6 | 9 | 5.7 | 17 | 2.6 |
|  | AG | 329 | 23.8 | 350 | 25.9 | 263 | 25.2 | 42 | 27.1 | 50 | 31.4 | 168 | 25.4 |
|  | GG | 1026 | 74.2 | 962 | 71.3 | 747 | 71.6 | 112 | 72.3 | 100 | 62.9 | 477 | 72.1 |
| rs6736233 |  |  |  |  |  |  |  |  |  |  |  |  |  |
|  | CC | 4 | 0.3 | 10 | 0.7 | 10 | 1.0 | 0 | 0.0 | 2 | 1.3 | 5 | 0.8 |
|  | GC | 163 | 11.8 | 203 | 15.0 | 153 | 14.6 | 22 | 14.4 | 32 | 20.1 | 93 | 14.1 |
|  | GG | 1216 | 87.9 | 1137 | 84.2 | 883 | 84.4 | 131 | 85.6 | 125 | 78.6 | 563 | 85.2 |
| rs1861270 |  |  |  |  |  |  |  |  |  |  |  |  |  |
|  | AA | 107 | 7.8 | 115 | 8.5 | 81 | 7.8 | 17 | 11.0 | 12 | 7.6 | 63 | 9.5 |
|  | AG | 538 | 39.0 | 525 | 38.9 | 423 | 40.5 | 54 | 35.1 | 61 | 38.6 | 260 | 39.3 |
|  | GG | 735 | 53.3 | 710 | 52.6 | 541 | 51.8 | 83 | 53.9 | 85 | 53.8 | 338 | 51.1 |
| rs6723097 |  |  |  |  |  |  |  |  |  |  |  |  |  |
|  | AA | 203 | 14.7 | 239 | 17.7 | 172 | 16.4 | 31 | 20.0 | 32 | 20.1 | 121 | 18.3 |
|  | AC | 653 | 47.2 | 638 | 47.2 | 514 | 49.1 | 63 | 40.6 | 80 | 50.3 | 315 | 47.6 |
|  | CC | 528 | 38.2 | 476 | 35.2 | 360 | 34.4 | 61 | 39.4 | 47 | 29.6 | 226 | 34.1 |
| rs2293554 |  |  |  |  |  |  |  |  |  |  |  |  |  |
|  | GG | 5 | 0.4 | 6 | 0.4 | 6 | 0.6 | 0 | 0.0 | 1 | 0.6 | 5 | 0.8 |
|  | TG | 185 | 13.4 | 206 | 15.3 | 153 | 14.7 | 25 | 16.1 | 38 | 23.9 | 97 | 14.7 |
|  | TT | 1193 | 86.3 | 1136 | 84.3 | 882 | 84.7 | 130 | 83.9 | 120 | 75.5 | 558 | 84.5 |
| rs1045485 |  |  |  |  |  |  |  |  |  |  |  |  |  |
|  | CC | 18 | 1.3 | 23 | 1.7 | 22 | 2.2 | 1 | 0.7 | 2 | 1.3 | 12 | 1.8 |
|  | GC | 256 | 19.1 | 251 | 19.0 | 197 | 19.3 | 29 | 19.3 | 25 | 16.2 | 127 | 19.5 |
|  | GG | 1065 | 79.5 | 1045 | 79.2 | 803 | 78.6 | 120 | 80.0 | 127 | 82.5 | 511 | 78.6 |
| rs1035140 |  |  |  |  |  |  |  |  |  |  |  |  |  |
|  | AA | 414 | 29.9 | 400 | 29.6 | 309 | 29.6 | 49 | 31.6 | 49 | 30.8 | 188 | 28.4 |
|  | TA | 674 | 48.7 | 646 | 47.8 | 503 | 48.1 | 68 | 43.9 | 78 | 49.1 | 315 | 47.7 |
|  | TT | 296 | 21.4 | 305 | 22.6 | 233 | 22.3 | 38 | 24.5 | 32 | 20.1 | 158 | 23.9 |
| rs700636 |  |  |  |  |  |  |  |  |  |  |  |  |  |
|  | AA | 267 | 19.3 | 265 | 19.7 | 197 | 19.0 | 31 | 20.0 | 31 | 19.6 | 138 | 21.0 |
|  | AC | 665 | 48.1 | 669 | 49.8 | 517 | 49.8 | 76 | 49.0 | 76 | 48.1 | 319 | 48.5 |
|  | CC | 450 | 32.6 | 410 | 30.5 | 324 | 31.2 | 48 | 31.0 | 51 | 32.3 | 201 | 30.5 |
| rs11679181 |  |  |  |  |  |  |  |  |  |  |  |  |  |
|  | CC | 428 | 31.0 | 437 | 32.4 | 334 | 32.0 | 50 | 32.3 | 47 | 29.6 | 223 | 33.8 |
|  | TC | 680 | 49.2 | 665 | 49.3 | 515 | 49.4 | 74 | 47.7 | 79 | 49.7 | 317 | 48.1 |
|  | TT | 274 | 19.8 | 248 | 18.4 | 194 | 18.6 | 31 | 20.0 | 33 | 20.8 | 119 | 18.1 |

Samples in which genotype was unknown are not included in this table.
