# Supplementary material for: Novel polymorphisms in caspase-8 are associated with breast cancer risk in the California Teachers Study
Source: BMC Cancer. 2016 Jan 12;16:14. doi: 10.1186/s12885-015-2036-9 (PMC4711015; doi:10.1186/s12885-015-2036-9)
Supplement: Additional file 2: — Table S2. SNPs from Lin et al. [26] that are in strong LD (r2 > 0.5) with rs2293554. *Odds Ratio (OR), Lower Confidence Limit (LCL), and Upper Confidence Limit (UCL) as reported in Lin et at. [26]. (DOC 44 kb) [file 12885_2015_2036_MOESM2_ESM.doc]

| Supplementary Table 2. SNPs from Lin et al. (29) that are in strong LD (r^2>0.5) with rs2293554 | | | | | | | |  |
| --- | --- | --- | --- | --- | --- | --- | --- | --- |
| chr 2 location | SNP | r^2 | D | D' | OR* | LCL* | UCL* | |
| 202138400 | rs7564683 | 0.795784 | 0.0616903 | 1 | 0.9366 | 0.8988 | 0.9759 | |
| 202138458 | rs7578639 | 0.778810 | 0.0615806 | 0.97903 | 0.9371 | 0.8993 | 0.9764 | |
| 202135429 | rs10186568 | 0.598384 | 0.0471749 | 1 | 0.9319 | 0.8911 | 0.9746 | |
| 202136595 | rs3817578 | 0.598384 | 0.0471749 | 1 | 0.9326 | 0.8918 | 0.9753 | |
| 202132672 | rs6749413 | 0.598384 | 0.0471749 | 1 | 0.9318 | 0.8908 | 0.9746 | |
| 202132107 | rs3754934 | 0.582231 | 0.0459653 | 1 | 0.9297 | 0.8886 | 0.9728 | |
| 202162657 | rs62191540 | 0.534040 | 0.0423364 | 1 | 0.9234 | 0.8813 | 0.9675 | |
| 202149696 | rs1045487 | 0.518065 | 0.0411268 | 1 | 0.9256 | 0.8840 | 0.9693 | |
| 202151781 | rs1045494 | 0.518065 | 0.0411268 | 1 | 0.9255 | 0.8842 | 0.9687 | |
| 202169155 | rs142683901 | 0.518065 | 0.0411268 | 1 | 0.9241 | 0.8820 | 0.9683 | |
| 202162406 | rs58659663 | 0.518065 | 0.0411268 | 1 | 0.9246 | 0.8825 | 0.9687 | |
| 202178675 | rs62189186 | 0.518065 | 0.0411268 | 1 | 0.9232 | 0.8813 | 0.9671 | |
| 202166592 | rs62191541 | 0.518065 | 0.0411268 | 1 | 0.9245 | 0.8825 | 0.9685 | |
| 202160116 | rs7561856 | 0.518065 | 0.0411268 | 1 | 0.9250 | 0.8829 | 0.9692 | |
| 202145359 | rs13422553 | 0.502135 | 0.0399172 | 1 | 0.9254 | 0.8836 | 0.9691 | |
| 202143491 | rs60928755 | 0.502135 | 0.0399172 | 1 | 0.9260 | 0.8840 | 0.9700 | |
| *Odds Ratio (OR), Lower Confidence Limit (LCI), and Upper Confidence Limit (UCI) as reported in Lin et at. (29) | | | | | | | |  |
